# Sources of Collagen for Biomaterials in Skin Wound Healing

**DOI:** 10.3390/bioengineering6030056

**Published:** 2019-06-30

**Authors:** Evan Davison-Kotler, William S. Marshall, Elena García-Gareta

**Affiliations:** 1 Biology Department, St. Francis Xavier University, Antigonish, NS B2G 2W5, Canada; 2 Regenerative Biomaterials Group, The RAFT Institute, Mount Vernon Hospital, Northwood HA6 2RN, UK

**Keywords:** collagen, collagen sources, recombinant collagen, biomaterials, regenerative medicine, tissue engineering, wound healing, skin

## Abstract

Collagen is the most frequently used protein in the fields of biomaterials and regenerative medicine. Within the skin, collagen type I and III are the most abundant, while collagen type VII is associated with pathologies of the dermal–epidermal junction. The focus of this review is mainly collagens I and III, with a brief overview of collagen VII. Currently, the majority of collagen is extracted from animal sources; however, animal-derived collagen has a number of shortcomings, including immunogenicity, batch-to-batch variation, and pathogenic contamination. Recombinant collagen is a potential solution to the aforementioned issues, although production of correctly post-translationally modified recombinant human collagen has not yet been performed at industrial scale. This review provides an overview of current collagen sources, associated shortcomings, and potential resolutions. Recombinant expression systems are discussed, as well as the issues associated with each method of expression.

## 1. Introduction

### 1.1. The Molecular Structure of Collagen

The 28 different collagen types of the collagen superfamily are further divided into eight subfamilies, with the majority of the collagen types belonging to the fibril-forming or fibrillar subfamily [1,2]. This subfamily includes collagen types I and III, the most abundant components of the extracellular matrix (ECM) of the dermal layer of skin. At a molecular level, collagens are characterized by repeated proline-rich amino acid sequences, with a glycyl residue appearing at every third position [1]. The repeated sequence is characterized as (Gly-X-Y)n, where X and Y can be any amino acid, although the X position is generally occupied by proline, and the Y occupied by 4-hydroxyproline. These polypeptides form α-chains, which assemble into a triple helix. Individual α-chains exist in a left-handed polyproline II-type helix, which then coil around each other to form a right-handed triple helix; this triple helix formation is also known as tropocollagen [2]. The collagen trimer forms around a central axis occupied by Gly residues; this positions the other X and Y residues on the outside of the triple helix. The helix may be formed by three identical α-chains (homotrimers), or two to three different α-chains (heterotrimers) [3]. Collagen types II, III, VII, VIII, and X are all formed from homotrimers, whereas collagen types I, IV, V, VI, IX, and XI are formed from heterotrimers. The combination of α-chains, as well as the peptide sequence, will dictate the type of collagen formed. Twenty-five α-chains have been discovered, leading to the assembly of the 28 different collagen types.

### 1.2. Collagen Stability

The abundance of collagen in animal systems, and particularly the fibrillar collagen type I, owes primarily to the unique mechanical and physiological characteristics, including thermal and chemical stability, mechanical strength, and physiological interactions. These parameters are derived from the complexity of the molecular and fibrillar structure at secondary, tertiary, and quaternary levels of protein organization. At both the secondary and tertiary level, intermolecular and intramolecular forces also promote stability. Left-handed α-chains are stabilized by interstrand hydrogen bonding, whereas the tertiary triple helix is stabilized by intra-strand n→π* interactions [4]. In an n→π* interaction, a nucleophile donates lone pair electron density to the empty π orbital of a nearby carbonyl group [5]. These tertiary fibril-forming collagens are initially synthesized as procollagen polymers, which undergo post-translational modification in the lumen of the endoplasmic reticulum. Propyl-4-hydroxylase and lysyl-hydroxylase hydroxylate procollagen proline and lysine residues, further contributing to molecular stability by preventing enzymatic degradation [3]. Once post-translationally modified procollagen is secreted from the cell, extracellular terminal N- and C- propeptides are cleaved from the ends of the procollagen molecules by metalloproteinases [6]. The newly-formed tropocollagen spontaneously aligns in longitudinally-staggered parallel strands, forming collagen fibrils [7]. 

### 1.3. Collagen in Skin and Wound Healing

Collagen is one of the most frequently used materials in protein-based scaffolds for skin wound healing; this is not only because of its sheer abundance in the body, but also because it is the main component of the dermal ECM. Specifically, collagen type I constitutes 80–85% of the dermal ECM, while collagen III constitutes 8–11%.

Several pro-regenerative physiological interactions are mediated by collagen during the complex wound healing process. Collagen contributes to hemostasis by interacting with platelets recruited to the wound site via chemotaxis [8]. The resulting clot provides a matrix for the influx of inflammatory cells. Platelets degranulate releasing alpha granules that secrete growth factors like epidermal growth factor (EGF), platelet-derived growth factor (PDGF), and transforming growth factor-beta (TGF-β). PDGF in conjunction with proinflammatory cytokines contribute to attract neutrophils to remove bacteria. TGF-β contributes to convert monocytes into macrophages, which initiate the development of granulation tissue and release various proinflammatory cytokines (e.g., interleukins 1 and 6 (IL-1, IL-6)) and growth factors (fibroblast growth factor (FGF), EGF, TGF-β, and PDGF). Proliferation of endothelial cells and subsequent angiogenesis is essential for the synthesis, deposition, and organization of a new collagen-rich ECM for infiltration of fibroblasts. Growth factors such as TGF-β and PDGF initiate phenotypic changes converting fibroblasts into myofibroblasts, which generate contraction forces that facilitate wound closure. Within hours of injury, reepithelialization starts with keratinocytes migrating over the provisional ECM. Once wound closure is achieved, keratinocytes undergo stratification and differentiation to restore the natural barrier provided by the skin. Collagen and elastin fibers replace the granulation tissue, followed by tissue remodeling, where new collagen is synthesized, and old collagen is broken down. The end product of the wound healing process is a scar. The success of this process depends on the coordination of growth factors, cytokines, and chemokines that effect cell behavior as a consequence of their binding to specific cell surface receptors or ECM proteins, of which collagen is the most abundant [8,9,10]. Thus, collagen is capable of interaction with a variety of regenerative pathways utilized in skin wound healing, ranging from angiogenesis to reepithelialization. Currently, collagen is used in a number of commercially available scaffolds for the purpose of skin wound healing, most notably Integra^®^ and Matriderm^®^ [11].

## 2. Sources of Collagen

The amino acid compositions of collagen types vary considerably between species, and these variations affect chemical and physical properties, thermal stability, solution viscosity, and crosslinking density [12,13,14]. Specific amino acid sequences in collagen act as a substrate for integrins, which are transmembrane receptors that facilitate cell-ECM adhesion. The integrin family of receptors consists of α and β subunits that form transmembrane heterodimers. The minimal integrin recognition sequence in collagens, when present as triple helical conformation, is GFOGER (O=hydroxyproline), which is recognized by α_1_β_1_, α_2_β_1,_ α_10_β_1_, and α_11_β_1_ integrins. While α_1_β_1_ and α_10_β_1_ preferentially bind to basement membrane type IV collagen, α_2_β_1_ and α_11_β_1_ integrins bind with higher affinity to collagen type I and other fibril-forming collagens [15]. In recent years, the important regulatory functions of the matrisome (the ensemble of ECM proteins and associated factors) has shed light on the many physiological functions of collagen that accompany its mechanical properties [16,17]. Given the binding specificity exhibited by many receptors, functional changes in the interaction of collagens will arise from structural differences in collagen derived from various animal and plant species. Collagen type I is the principal type used in scientific research because of its relatively high abundance and low cost. Rat tail tendon, porcine, and bovine skin-derived collagen type I are most popular, although alternatives such as human placenta and human skin-derived collagen type I are available at a higher cost [14]. Collagen type III has traditionally been isolated from bovine placenta, human placenta, human skin, chicken skin, and rat skin, although alternatives such as marine-derived and recombinant sources have been used. 

Although animals make up the majority of collagen sources used in biomaterial science, animal-derived collagens are not without their risks. Variance in purification strategies results in a risk of infectious disease transmission, as observed in the case of bovine spongiform encephalopathy transmission via prion-contaminated bovine-derived scaffolds [13]. Allergy and immunogenicity pose additional problems—clinical observations have indicated that 2–4% of the population is allergic to porcine and bovine-derived collagen [18,19]. The negative outcomes associated with animal-derived collagen are prevented by the use of human-derived collagens; however, the biophysical profile of human placental and skin-derived collagens is affected by ethnicity, age, environmental setting, and genotypes of the source tissue, resulting in variability among samples [20]. For example, increased intermolecular crosslinking has been observed with age, and is thought to be responsible for the increased resistance to collagenase, decreased elasticity, and loss of osmotic swelling capacity of aged collagen in vivo [18,19,21]. Whereas the natural enzymatically-formed aldimine types are dissociated by application of a dilute acid, age-related ketoimine types are not [22]. Extraction processes used in animal and human collagen isolation also produce inter- and intramolecular crosslinking, which result in as much as 30% of collagen mass attributed to molecules other than the α and β subunits [21,22]. The increase in crosslinking results in collagen with poor water solubility and a reduced capacity to form a structured matrix [22]. The mechanical and chemical variation of human-derived collagen, coupled with the potential for infection, immunogenicity, and batch-to-batch variability in animal-derived collagen, have led to an increased interest in alternatives such as recombinant collagen. Recombinant human collagen (rhCOL) biosynthesis offers the potential for consistent, biochemically-identical collagen production at scale. Recombinant collagen expression models have been successfully demonstrated in both prokaryotic and eukaryotic cells, including *Escherichia coli*, fungus, plant, and animal-based systems [23]. The creation of high protein throughput models has presented as one of the most difficult hurdles to surmount, as well as the addition of post-translational modifications to collagens synthesized in recombinant systems lacking native propyl 4-hydroxylase activity.

### 2.1. Bovine Sources

Bovine-derived collagen type I is generally isolated from the Achilles tendon, although bone and skin have also been used as source tissues [24,25]. Bovine-derived collagen type III is solely isolated from the skin for use in research, although alternative tissue sources exist [26]. As mentioned, variation in isolation protocols and source tissue often leads to batch-to-batch inconsistency, as well as the potential for transmissible disease contamination. The transmission of both bovine spongiform encephalopathy and viral vectors to humans is possible through the use of bovine-derived collagen, indicating that the source population and purification methods are important considerations. Even if the risk of pathogenic contamination was eliminated, the issue of consistency across manufacturers would persist. Variation in consistency depends on age, region of origin, and genetic inheritance. The amount of collagen recovered during isolation varies according to the age of the bovine tissue, with younger tissues yielding more collagen [24]. In addition, proteoglycan distribution and post-translational modification also varies with age and has the potential to modulate the thermal stability and fibrous self-assembly of tropocollagen [24,27,28]. Proteoglycans, such as decorin, play a role in regulating fibril formation by sterically hindering binding to collagen [29]. Specific dermatan sulfate proteoglycans derived from bovine tendon also appear to inhibit fibrillogenesis of collagen type I; therefore, researchers are encouraged to take the age of the bovine donor population into account in an attempt to anticipate variation in the biochemical composition of the extract [30,31]. Bovine collagen exhibits positive characteristics of biocompatibility and low immunogenicity—in general, it is well-tolerated in vivo, and does not elicit an immune response in most people, except those with a significant collagen allergy [32,33]. 

### 2.2. Rodent Sources

Rat-tail tendon (RTT) is one of the most commonly used sources of collagen type I among researchers (in contrast to industrial use), given the extensive amount of literature concerning isolation and characterization. RTT contains 90–95% collagen type I by weight; the high proportion of collagen ensures significant yields upon isolation [34,35]. Researchers investigated the changes in mechanical characteristics of tendon segments in relation to age and found that, as the rat ages, the collagen becomes less elastic and more resistant to force [35]. Unsurprisingly, the level of collagen crosslinking also affects the stress–strain curve (tangent moduli), with crosslinked RTT-derived collagen scaffolds exhibiting a substantially higher tangent modulus than non-crosslinked scaffolds. Given that the keto-imine crosslinks formed as a result of age do not undergo the same disassociation during acid extraction as do aldimine linkages, variation in the age of the source rat population is expected to result in variation in crosslink density post-isolation, and; therefore, differences in the mechanical properties of manufactured scaffolds [22,36]. While RTT collagen type I is widely used in research, it is not used in clinical products as medical-grade RTT collagen I is not available. 

### 2.3. Fish, Mollusc, and Marine Invertebrate Sources

Collagen type I derived from marine sources has become an increasingly popular subject of investigation over the past decade. Fish collagens are advantageous because of a reduced risk of disease transmission and the abundance of unused collagen-containing tissues in the food industry. Marine-derived collagen has been successfully isolated from fish skin, scales, and bone, as well as various invertebrate tissues [14,37,38,39]. During food processing and whole-fish preparation, the muscle tissue is generally the only portion of the fish harvested for human consumption. Other tissues, including the head, skin, scales, internal organs, and bones are made into fish meal food in aquaculture. Given the extensive use of collagen in biomaterial production, the repurposing of fish and other marine animal by-products has the potential to generate substantial value. Marine-derived collagen demonstrates chemical and mechanical properties that vary from those observed in mammalian collagen; variation includes lower melting point, lower viscosity at a given concentration in solution, lower water solubility, higher fibrillar proportions of glutamic acid and alanine, and lower proportions of proline [37,40]. The biological properties of marine-derived collagen in relation to tissue engineering and biomaterials are also quite favorable—scaffolds composed of collagens isolated from marine sources demonstrate high biodegradability (allowing the body to replace the scaffold with regenerated tissue over time), low immunogenicity, and high biocompatibility [40,41,42]. It should be noted; however, that discrepancies between marine and mammalian collagen interactions in vitro were reported. Rastian et al. isolated type I collagen from the jellyfish *Catostylus mosaicus* via acid extraction, noting several clear differences between acid solubilized collagen (ASC) I from rat tail (RASC) and jellyfish (JASC) [43]. JASC was characterized by a reduction in melting point, as well as lower viscosity and proline content. The proportion of α1 to α2 chains was approximately equal to that observed in native human collagen, and, importantly, both RASC and JASC promoted cellular attachment and proliferation when manufactured as an agarose-blended scaffold [43]. Other invertebrates such as squid have also been studied as potential sources of collagen for biomaterial and wound healing purposes. Wichuda et al. analyzed both the amino acid composition and physical characteristics of acid-soluble squid-derived collagen (SASC) compared to JASC [44]; both SASC and JASC exhibited high solubility at moderately acidic pH ranges (pH 3–5). However, the level of solubility dropped markedly in the presence of NaCl. Finally, the amino acid composition of both extracted collagens was characterized as slightly different when compared to RASC; the variance in amino acid composition likely accounts for variability in solubility, melting point, and viscosity [44]. 

In addition to scaffold formation, JASC has been investigated for use as a hemostatic (blood coagulation promoter) agent; Cheng et al. characterized crosslinked, lyophilized collagen sponges extracted from the mesoglea of *Rhopilema esculentum* as an effective hemostat when compared to medical gauze, noting significant platelet and red blood cell adherence to the collagen matrix [45]. It should be noted that the nature of lyophilization as a scaffold formation method induces a gradient porous structure, which is highly effective at absorbing material and inducing adherence [45,46,47]. Although Cheng et al.’s research characterizes JASC as more effective than medical gauze, the adhesion tests revealed the hemostatic mechanism as mainly physical absorption, failing to position JASC as a superior hemostat when compared to traditional collagen or fibrin hemostatic agents [45]. 

Fish-derived collagen exhibits amino acid sequences with reduced variability when compared to JASC or SASC; however, significant variations in denaturation temperature have been observed across species [13,14,39,48]. Sun et al. measured the denaturation temperature of collagen I isolated from pacific cod (*Gadus microcephalus*) at 14.5 °C, which is significantly lower than that of mammalian-derived collagen. Collagen isolated from grass carp (*Ctenopharyngodon idellus*); however, did not denature until heated to 33 °C [49]. These variations in temperature are likely due to differences in hydroxyproline content, as Hyp is known to thermodynamically stabilize the collagen triple helix, and interspecies variation has resulted in comparative changes to collagen thermostability [50]. Several other studies have measured the conformational changes of fish-derived collagen at various temperatures and are summarized in Table 1. The variability in denaturation temperature of fish-derived collagen is likely determined by variation in Gly-Pro-Hyp sequences [50]. While the noted variation does not preclude marine-derived collagen from use in biotechnology, collagens with a denaturation temperature significantly lower than the human core body temperature of 37.5 °C will likely necessitate significant additional crosslinking if they are to be used in wound healing or other biomaterial applications. 

### 2.4. Recombinant Expression Systems

The issues that afflict extracted collagen—namely heterogeneity across species and intraspecies demographics, potential for pathogen transfer, and immunogenicity—have prompted the investigation of alternative synthetic methods. Recombinant human collagen (rhCOL) has emerged as a potentially viable production method, eliminating the risk of interspecies variance and pathogenic infection, while ensuring homogeneity across batches [57]. Early attempts to produce rhCOL in prokaryotic and yeast systems yielded short and unstable fibers; both prokaryotes (e.g., *E. coli*) and yeast species (e.g., *Pichia pastoris*) lack the necessary enzymatic machinery to produce post-translationally modified collagen, owing to the absence of hydroxylases (Table 2). Combinations of viral and human hydroxylase transduction has; however, yielded hydroxylation of collagen residues similar to those observed in native collagen [58,59].

#### 2.4.1. Prokaryotic Expression Systems

##### *E. Coli* 

*Escherichia coli* is the most commonly used transgenic organism for the production of proteins through recombinant expression systems, owing to the breadth of research on *E. coli* genomic composition and outcomes of modification. The rapid growth rate exhibited by *E. coli* in culture also positions it as an ideal system for industrial-scale recombinant protein production [60]. *E. coli* naturally produces a collagen-like protein characterized by Gly-X-Y sequence repeats and a C-terminal trimerization domain; however, the protein lacks the characteristic proline hydroxylation of human collagen [61]. The post-translational modifications to collagen expressed by human cells are crucial to the thermostability of the protein; unhydroxylated collagen exhibits significantly reduced helical propagation and inhibited fiber self-assembly, reducing the usefulness of bacteria-expressed collagen for human applications [62,63]. The lack of post-translational modification in proteins synthesized by *E. coli* has resulted in increased investigation of eukaryotic expression systems, although production yields have been low [64]. Recently, the issue of hydroxylation in bacterial collagen expression has been resolved via the transduction of viral hydroxylase isolated from the giant aquatic *Mimiviridae* family of viruses [58]. Rutschmann et al. transduced viral lysyl hydroxylase L230 and propyl 4-hydroxylase L593 into the *E. coli* expression system, yielding hydroxylation frequencies similar to that observed in natural human-derived collagen [58]. While the expressed collagen is characterized by similar hydroxylation levels compared to native collagen, the amino acid sequences differ significantly from human collagen; this variance has the potential to result in altered binding to proteins and membrane receptors and increase the immunogenicity of the recombinant collagen [65]. Several collagen-like polymers (CLPs) have been recombinantly expressed by *E. coli* and exhibit thermostability, despite the lack of post-translational hydroxylation [66]. Issues of CLP overproduction in *E. coli* systems have led to the assembly of misfolded and aggregated insoluble collagens; this abnormal synthesis pattern has been improved via the identification and expression of exogenous chaperone proteins [67]. The expressed CLPs are easily modified and have the potential to initiate research concerning the customization of binding motifs within the collagen triple-helix. Given the current issues surrounding hydroxylation and amino acid sequence parity, collagen produced by *E. coli* and similar bacterial systems is best suited for mechanical applications, rather than those relating to physiological interactions. 

#### 2.4.2. Eukaryotic Expression Systems

##### Yeast

Species of yeast have been used in recombinant protein production since the 1980s, when the human gene for interferon was transduced within a *Saccharomyces cerevisiae* cell [68]. Since then, *S. cerevisiae* and *Pichia pastoris* have been frequently used as recombinant expression systems for a variety of proteins. In 2009, 19% of all protein-based recombinant pharmaceuticals were synthesized within an *S. cerevisiae* expression system [69]. The persistence of yeast as a model recombinant system is due to the characteristic rapid growth rates and ease of genetic modifications, coupled with the ability to synthesize enzymes capable of post-translational modification and protein folding. Although yeast lacks native propyl 4-hydroxylase (P4H), the transduction of P4H α and β subunits together with a collagen-coding gene results in the production of hydroxylated collagen fibrils [70]. Recombinant human collagen (collagen genes COL1A1 and COL3A1) synthesized by yeast is more similar to native collagen than that produced by *E. coli* systems; however, the collagen is only expressed as a homotrimer, and the transduced cDNA is often fragmented [71,72]. For collagens that exist as a homotrimer, such as collagen III, recombinant production via yeast transduction has been used in hemostatic applications, successfully eliciting platelet adhesion, aggregation, and growth factor production [73]. The elimination of lot-to-lot variance and human origin positions rhCOL type III as a superior hemostatic agent when compared to similar products, such as bovine-derived Avitene™ [74,75]. Pharmaceutical labelling specifically warns against the use of Avitene™ in patients sensitive to bovine-derived collagen, because of the detectable levels of intercalated bovine serum protein within the matrix [74,75]. The use of rhCOL type III not only reduces the risk of allergic reaction, but also eliminates the possibility of pathogenic infection due to the animal source. Advances in genetic manipulation have vastly improved the yields and quality of collagen produced by yeast expression systems, leading many companies to adopt the production method on an industrial scale. Companies such as FujiFilm and FibroGen have synthesized rhCOL peptides using both *S. cerevisiae* and *Pichia pastoris*, and the resulting hydrogels have been used for a variety of applications ranging from wound healing to optically clear corneal scaffolds [76,77]. 

##### Plant

Several plant systems have been used to express rhCOL type I with relatively positive outcomes. P4H genes were successfully transduced into both maize (*Zea mays*) and tobacco plants (*Nicotiana tabacum*), yielding plant cells capable of producing collagen I homotrimer helices [63,78,79,80]. Additionally, both collagen I α and β strands have been expressed in a transgenic tobacco plant transduced with P4H and lysyl hydroxylase 3 (LH3) enzyme-encoding genes, yielding post-translationally modified heterotrimeric collagen [81]. The collagen exhibited thermostability and resistance to protease activity up to 39 °C, similar to that expressed by native human collagen. The amino acid sequence of the rhCOL type I expressed by transgenic tobacco plants was 100% similar to native human procollagen I, standing in stark contrast to the variance exhibited by *E. coli*-expressed rhCOL [81,82]. The recombinant collagen was also capable of supporting cellular adhesion, binding and proliferation of cultured adult peripheral blood-derived endothelial progenitor-like cells; the successful interaction with vascular endothelial cells indicate that collagen matrices made from this material may be ideal for use in wound repair, as angiogenesis is a crucial step in the regeneration of skin [81,83]. Recently, rhCOL type I has been combined with platelet-rich plasma to create a flowable wound-healing gel [84]. Upon injection into a wound site, the gel forms a collagen-fibrin matrix capable of triggering cellular recruitment, adhesion and proliferation [85]. The addition of platelet-rich plasma results in platelet-derived growth factor and vascular endothelial growth factor production within the matrix, further enhancing the proliferation of fibroblasts and vascular recruitment [84,85,86]. In vivo research indicates that wounds treated with rhCOL type I synthesized by a tobacco recombinant system significantly accelerate wound closure, when compared to animal-derived collagen flowable gels [84]. Histological investigation revealed earlier reepithelialization and inflammation in rhCOL-treated wounds, followed by denser infiltration of vasculature; these results are consistent with an earlier and enhanced wound healing response when compared to current treatment methods [84]. 

##### Insect

Both whole insect organisms and cultured cells have been transfected with COL1A1 and COL3A1 genes with variable success. Tomital et al. elicited the production of rhCOL type III within the cocoon of a transgenic silkworm by injecting silkworm eggs with the vectorized cDNA [87]. Preliminary data indicated that the C-propeptide domain of the rhCOL type III suppressed expression of the fusion cDNA; therefore, researchers transduced fusion cDNA with a deleted C-propeptide domain and on additional fibroin light chain into the eggs via the piggyBac transposon vector system [87]. Given the high protein content of cocoons (>95%), rhCOL type III production within the cocoon could potentially act as a scalable expression system. Upon conformational analysis; however, the protein was revealed to contain very low proline hydroxylation, and therefore low protease resistance and decreased thermostability [87]. Additional data indicates that the activity of P4H in silk glands is very low, contributing to poor post-translational modification; therefore, the addition of P4H to the transduction vector has the potential to result in the expression of hydroxylated rhCOL type III. However, future research is necessary to confirm this hypothesis. Silk fibroin derived from silk worms has previously been used in conjunction with recombinant collagen derived from an *E. coli* expression system, although the collagen-like protein was only characterized by Gly-X-Y repeats, rather than exhibiting characteristics indicative of a single collagen type [88]. The addition of recombinant collagen significantly enhanced the proliferation and survival of fibroblasts when compared to pure fibroin cultures, demonstrating a potentially useful application of the aforementioned collagen-like proteins produced by prokaryotic expression systems [88]. 

Isolated insect cells have also been used as rhCOL expression systems. Clonal High Five™ (HF) insect cells derived from parental cabbage looper (*Trichoplusia ni*) and produced by Invitrogen are commonly used for the expression of recombinant proteins [89]. Recombinant human collagen III produced within the HF cells in the absence of recombinant P4H resulted in a considerable amount of intracellular 4-hydroxyproline production; however, the resulting rhCOL type III was not sufficiently unhydroxylated, and exhibited low thermostability [90]. The addition of recombinant P4H and ascorbate in the culture medium was found to increase collagen hydroxylation and re-establish thermostability; these results indicate that insect cells are similar to fibroblasts in terms of their dependence on ascorbate to produce correctly processed and folded collagen molecules [87,90]. 

##### Mammals and Cultured Human Cells

Given the folding and post-translational modification issues associated with non-mammalian cells, the logical evolution of recombinant protein expression is the transduction of collagen-encoding genes into a human cell line that does not normally produce the target collagen. Transgenic mammals have successfully produced rhCOL type I; for example, Toman et al. were able to transduce COL1A1 gene within mouse embryos, resulting in large amounts of rhCOL produced from the mammary glands [91]. These heterotrimeric proteins were correctly folded, processed and secreted in a soluble form. Additionally, collagen VII has been transduced within the ovaries of the Chinese hamster [92]. Once extracted, the rhCOL type VII was intravenously administered to mice with recessive dystrophic epidermolysis bullosa, a disease of the epidermal-dermal junction; the treatment successfully reversed the disease phenotype in affected mice by localizing within the tissues of the epidermal-dermal junction, and acting as anchoring points for cells of both tissue layers [93,94]. Several human cell lines, including fibrosarcoma cells (HT1080) and embryonic kidney cells (293-EBNA), have also successfully produced rhCOL type I, V, and VII in culture (Table 2) [93,94,95,96]. Although most rhCOL produced in cultured human cells is identical to that produced in vivo, cultured cells generated relatively low collagen yields when compared to the majority of the aforementioned expression systems [95]. Thus, transgenic mammals could potentially act as a high-yield source of recombinant human collagen; however, the low yields produced by cultured mammalian cells is insufficient to act as a rhCOL expression system at an industrial scale.

## 3. Conclusions and Future Directions

The issue of a source for collagen is one which persists beyond the scope of this paper. Animal-derived collagens used in medicine continue to pose immunogenic and pathogenic risks, although incidences of pathogenic infection resulting from scaffold implantation are rare. Recombinant DNA technology could potentially result in the production of human collagen at industrial scales; however, issues surrounding post-translational modification, protein folding, and batch yields reduces the likelihood of this technology becoming mainstream in the immediate future. Still, recombinant technology is positioned as a useful solution to the issue of isolating less abundant collagen types such as collagen VII, the injection of which can result in the resolution of pathologies, such as those affecting the epidermal–dermal junction. At this point, *E. coli*-produced rhCOL is error-prone, and mammalian cell rhCOL yields are too low; recombinant plant collagen; however, has the potential to excel as a recombinant system due to its scalability and high-quality post-translationally-modified collagen. Totaled, recombinant technology is by far the superior source of collagen; however, issues relating to scalability will hinder widespread industrial adoption until these difficulties are resolved.

## Figures and Tables

**Table 1:** Summary of marine-derived collagen isolates, with notes indicating variation from mammalian-derived collagen and potential applications.

	Source	Expressed Collagen	Notes	Reference
Marine Invertebrates	Jelly blubber (*Catostylus mosaicus*)	Type I	Low denaturation temperature; reduced viscosity and proline content compared to RASC	[43]
	Flame jellyfish (*Rhopilema esculentum*)	Type I	Collagen sponge used as hemostat; effective due to physical properties, no noted superiority to traditional protein-based hemostatic agents	[45]
	Atlantic sea nettle (*Chrysaora quinquecirrha*)	Type I	High thermal denaturation temperature (37 °C); large variance in amino acid content compared to RASC; significant amount of hydroxyproline	[51]
	Barrel jellyfish (*Rhizostoma pulmo*)	Type I	Heparin inhibited cellular adhesion to jellyfish-derived collagen by 55%; fibrillar morphology similar to mammalian collagen	[37]
	Squid (*Doryteuthis singhalensis*)	Type I	High thermal denaturation temperature (35 °C), indicating potential for commercial use	[52]
	Bigfin reef squid (*Sepioteuthis lessoniana*)	Type I	Variance in amino acid composition compared to RASC; high solubility at narrow acidic pH range 4–5	[44]
	Crown-of-thorns starfish (*Acanthaster planci*)	Type I	Denaturation temperature of 33 °C, comparable to mammalian collagen; proline content similar to mammalian collagen	[53]
Teleost Fish	Atlantic salmon (*Salmo salar*)	Type I	Fish skin collagen less resistant to high temperatures, with lower denaturation and thermal decomposition temperatures being observed in fish skin collagen compared to bovine-derived collagen	[13]
	Pacific cod (*Gadus macrocephalus*)	Type I	Proline and hydroxyproline content lower than bovine- and porcine-derived collagen; extremely low thermal denaturation temperature (14.5 °C), likely not useful for biomaterials without significant crosslinking	[42]
	Olive flounder (*Paralichthys olivaceus*)	Type I	Significant collagen extraction yield from skin	[41]
	Catfish (*Tachysurus maculatus*)	Type I	Type I collagen extracted from the swim bladder and used to form chitosan scaffold; crosslinking with glutaraldehyde yielded a scaffold with high tensile strength, low antigenicity, and high thermal stability	[54]
	Nile tilapia (*Oreochromis niloticus*)	Type I	Tilapia-derived collagen sponges rarely elicited an inflammatory response in vivo, statistically similar to those elicited by bovine-derived collagen	[55]
	Chum salmon (*Oncorhynchus keta*)	Type I	Very low denaturation temperature (18.6 °C), indicating a necessity to crosslink if used in biomaterials	[48]
Elasmobranch Fish	Blacktip shark (*Carcharhinus limbatus*)	Type I	Denaturation temperature (34 °C) similar to that of mammalian-derived collagen	[56]

**Table 2:** Summary of recombinant expression systems, with notes indicating variation from mammalian-derived collagen, methodological issues, and potential applications.

	Expression System	Transduced Gene	Expressed Collagen	Notes	Reference
Prokaryote	*Escherichia coli*	COL1A1	Type I	Different amino acid expression when compared to natural collagen	[82,97]
	*Escherichia coli*	COL3A1; L230, L593 (APMV)	Type III	Expression collagen III and *mimivirus* propyl and lysyl hydroxylases yielded hydroxylation levels similar to those expressed in humans	[58,59]
Yeast	*Pichia pastoris*	COL1A1, PH4A/B	Type I	-	[60,76,98]
	*Pichia pastoris*	COL3A1, PH4A/B	Type III	Recombinant hydroxylated collagen III exhibited hemostatic properties in vivo	[70,72,73]
	*Saccharomyces cerevisiae*	COL3A1, PH4A/B	Type III	Computational algorithm determined optimal oligonucleotide sequence	[99]
				Addition of non-native cysteine residues created crosslinking and anchoring sites; increased melting point compared to other RHC	[100]
Plant	*Nicotiana tabacum*	COL1A1/2, P4HA/B, LH3	Type I	Expressed triple helix similar to native collagen; supported growth and proliferation of vascular endothelial cells	[81]
	*Zea mays* seed	COL1A1, P4HA/B	Type 1	High yield collagen I produced by recombinant corn seed; hydroxylation of collagen led to enhanced thermostability.	[79,80]
Human cell lines	HT1080 fibrosarcoma cells	COL1A1	Type I	BP loss during initial propagation in *E. coli* necessitated reconstruction via PCR; recombinant expression produced over-modified pro 1(I) chains	[85]
	HEK 293 kidney epithelial cells	COL5A1	Type V	Addition of ascorbate to medium resulted in correctly folded, stable triple helix	[96]
	HEK 293 kidney epithelial cells	COL7A1	Type VII	Anchoring type VII collagen used to treat dystrophic epidermolysis bullosa, by establishing dermal-epidermal adherence	[92,93,94]
Mammal	*Mus Musculus* (Mammary gland)	COL1A1	Type I	Soluble (1)_3_ (I) procollagen with post-translational proline and lysine hydroxylation secreted in milk	[91]
Insect	*Spodoptera frugiperda* (Sf9 cells)	COL3A1	Type III	Hydroxylated triple helix molecules expressed intracellularly	[90]
	*Trichoplusia ni* (High Five, Invitrogen)	COL3A1	Type III	Hydroxylysine residue content slightly lower than non-recombinant expression	[90]
	*Bombyx mori*	COL1A1	Gly-X-Y collage-like homodimer	Amino acid sequence and contents varied from natural collagen	[88]
